# The Effectiveness of Ultrasound-Guided Steroid Injection Combined with Miniscalpel-Needle Release in the Treatment of Carpal Tunnel Syndrome vs. Steroid Injection Alone: A Randomized Controlled Study

**DOI:** 10.1155/2019/9498656

**Published:** 2019-02-24

**Authors:** Subo Zhang, Fei Wang, Songjian Ke, Caina Lin, Cuicui Liu, Wenjun Xin, Shaoling Wu, Chao Ma

**Affiliations:** ^1^Department of Rehabilitation Medicine, Sun Yat-sen Memorial Hospital, Sun Yat-sen University, Guangzhou, Guangdong Province, China; ^2^Department of Neurology of The First People's Hospital of Jiashan, Jiaxing, Zhejiang Province, China; ^3^Department of Physiology and Pain Research Center, Guangdong Province Key Laboratory of Brain Function and Disease, Zhongshan Medical School, Sun Yat-sen University, Guangzhou, Guangdong Province, China

## Abstract

**Objectives:**

Carpal tunnel syndrome (CTS) is one of the most common nerve entrapment syndromes, which has a serious impact on patients' work and life. The most effective conservative treatment is steroid injection but its long-term efficacy is still not satisfactory. The aim of this study was to evaluate the effectiveness of steroid injection combined with miniscalpel-needle (MSN) release for treatment of CTS under ultrasound guidance versus steroid injection alone. We hypothesized that combined therapy could be more beneficial.

**Methods:**

Fifty-one patients with CTS were randomly allocated into two groups, namely, steroid injection combined with MSN release group and steroid injection group. The therapeutic effectiveness was evaluated using Boston Carpal Tunnel Questionnaire (BCTQ), cross-sectional area (CSA) of the median nerve, and four electrophysiological parameters, including distal motor latency (DML), compound muscle action potential (CMAP), sensory nerve action potential (SNAP), and sensory nerve conduction velocity (SNCV) at baseline, 4 and 12 weeks after treatment.

**Results:**

Compared with baseline, all the parameters in both groups showed statistically significant improvement at week 4 and week 12 follow-up, respectively (*P*<0.05). When compared with steroid injection group, the outcomes including BCTQ, DML, CMAP, SNCV, and CSA of the median nerve were significantly better in steroid injection combined with MSN release group at week 12 after treatment (*P*<0.05).

**Conclusions:**

The effectiveness of steroid injection combined with MSN release for CTS is superior to that of steroid injection alone, which may have important implications for future clinical practice. This Chinese clinical trial is registered with ChiCTR1800014530.

## 1. Introduction

Carpal tunnel syndrome (CTS) is the most common and widely studied nerve entrapment syndrome, accounting for 90% of all such disorders [1]. It is caused by compression of the median nerve as it travels through the wrist at the carpal tunnel [2]. Patients with CTS mainly experience pain and paresthesias in the distribution of the median nerve, which includes the palmar aspect of the thumb, index and middle fingers, and radial half of the ring finger [3]. This syndrome often brings serious problems to patients' life and work.

Conservative treatment includes physical therapy such as splinting and application of systemic or local anti-inflammatory drugs [4]. Among them, local injection of steroid is a very classic and commonly used strategy [5, 6]. Steroid injection exerts its function mainly through reducing edema to improve the spatial relation between the carpal tunnel and the median nerve and tendons [7]. However, studies have reported that steroid injection is not as effective as surgical decompression, especially in the long term. Even if steroid injection temporarily improves symptoms in some patients with CTS, it does not completely obviate the long-term need for surgery [8–11]. This may be due to the fact that injections fail to directly release and decompress the carpal tunnel.

The miniscalpel-needle (MSN), developed in China, is a medical instrument similar to acupuncture needle, which can release transverse carpal ligament. So it can achieve the effect of surgical release to some extent but more minimally invasive. Treatment with MSN has been reported to relieve the symptoms of various myofascial syndromes such as chronic neck pain, plantar fasciitis, and gluteus medius calcific tendonitis without any obvious side effects [12–15]. Our previous studies also showed that MSN release was effective in treating trigger thumb [16] and trigger points in the upper trapezius muscle [17]. Hence, based on its mechanical loosening and acupuncture functions, whether steroid injection combined with MSN release can be more effective in treating CTS deserved further exploration.

The technology of ultrasound (US) guided injection has been gradually used in treating several conditions, including hip osteoarthritis [18], lower lumbar radicular pain [19], intraarticular knee injection [20], etc. Recent studies have shown that US-guided steroid injection may be more effective than blind injections in treating CTS [21, 22]. In our study, we performed both MSN release and steroid injection under the guidance of ultrasound to ensure the precision of the treatment. The aim of our study is to compare combined therapy of steroid injection and MSN release with simple steroid injection for the treatment of CTS under ultrasound guidance.

## 2. Materials and Methods

### 2.1. Patients

Patients with symptoms of pain, numbness, or tingling in the median nerve distribution area of hand visited Department of Rehabilitation Medicine, Sun Yat-sen Memorial Hospital, from February 2016 to May 2017. After confirmation by physical and electrophysiological inspection, 51 patients (51 wrists) meeting the following criteria were recruited [23]: (1) pain, numbness, or tingling in the median nerve distribution area of hand, (2) nocturnal worsening of the symptoms, (3) positive Tinel and/or Phalen sign, (4) a slower median nerve conduction (SNCV≦50 m/s and/or DML≧4 ms), (5) patients with unilateral disease, and (6) the desire of the participant to have either a steroid injection or steroid injection plus MSN release. Patients were excluded from this study for the following: (1) symptomatic CTS because of diabetes, thyroid disease, or rheumatic disease, (2) cervical radiculopathy or other polyneuropathy, (3) age<18 years, (4) pregnancy, (5) steroid injection for CTS in the preceding 6 months, (6) history of wrist fracture, (7) prior carpal tunnel decompressive surgery, (8) the presence of infection or skin lesion at the site of injection, (9) patients with bilateral disease, and (10) refusal of informed consent or inability to participate in follow-up. Fifty-one patients with unilateral disease were randomly assigned to steroid injection combined with MSN release (Group A) or steroid injection (Group B). A random number table was generated by computer and the random numbers were divided into two groups, with odd numbers into Group A and even numbers into Group B. We wrote the random number and the allocation result in sealed numbered envelopes orderly, only to open one once a patient has been recruited and consented. Finally, Group A had 25 patients (25 wrists) while Group B had 26 patients (26 wrists). The demographic data of both groups are shown (Table 1). In this study, all the participants received written informed consent and the study protocol was approved by local ethics committee (Medical ethics committee of Sun Yat-sen Memorial Hospital, Sun Yat-sen University) and registered in the Chinese Clinical Trial Registry (ChiCTR1800014530).

### 2.2. US-Guided Steroid Injection and MSN Release

Group A was treated with US-guided MSN release firstly so as to release the nerve entrapment. Immediately after that, steroid injection was performed. Group B was treated only with US-guided steroid injection. The US-guided injection was conducted using out-of-plane approach while MSN release was conducted using inplane approach. After treatment, in addition to proper hand movements, any other complementary or alternative treatment was not allowed during the 12-week follow-up. All of the MSN release and steroid injection operations in this study were performed by an experienced senior doctor.

**Figure 1:**
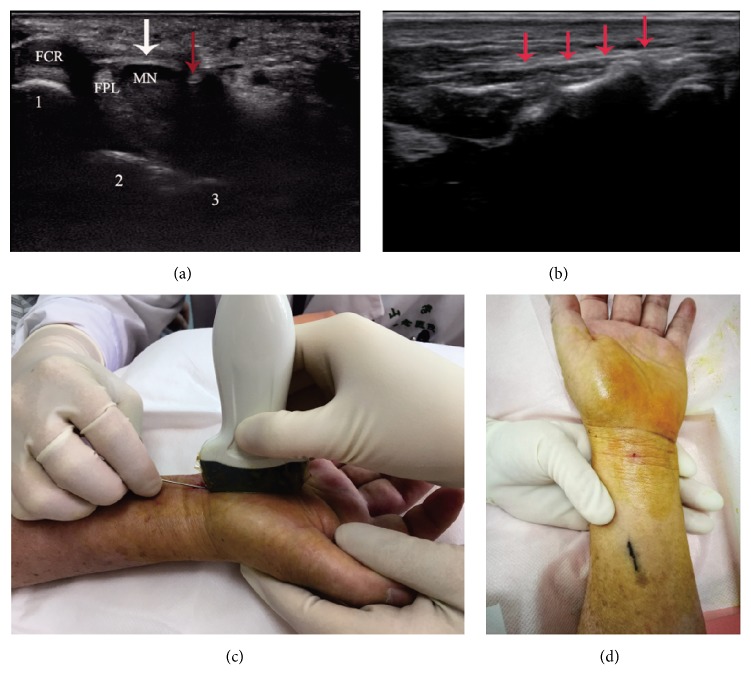
The procedures of steroid injection and MSN release. (a) Sonogram showing steroid injection using out-of-plane method in a 41-year-old woman. The white arrowhead refers to the transverse carpal ligament. The red arrowhead refers to the needle tip. MN: median nerve. FCR: flexor carpi radialis. FPL: flexor pollicis longus. 1: scaphoid bone. 2: lunate bone. 3: triquetral bone. (b) Sonogram of MSN release using inplane method in a 51-year-old man. The red arrowheads refer to the MSN shaft which is seen as a hyperechoic bright line. (c) Operation diagram of MSN release in a 55-year-old woman showing the position of the transducer and the MSN during inplane method for carpal tunnel decompression. (d) When the MSN was withdrawn, only a pinhole can be seen in the patient's wrist.

US-guided steroid injection was performed based on previous clinical report [22, 24, 25]. Patients sat in a chair, with the forearm and wrist supinated in a slight dorsiflexion position to better expose the carpal tunnel. After skin antisepsis, a transducer was placed vertically around the distal wrist crease to observe the overall situation of the carpal tunnel and finally maintained perpendicular to the median nerve. Under guidance of US, a 25-gauge needle was introduced into the carpal tunnel radial or ulnar to the median nerve. Because we used the out-of-plane approach, only the needle tip was identified as a moving reflector in ultrasonic imaging. After confirming that the needle tip was in the carpal tunnel, 1.0 ml of compound betamethasone (2 mg betamethasone sodium phosphate and 5 mg betamethasone dipropionate) together with 1.0 ml of 1% lidocaine was injected around the median nerve (Figure 1(a)). Then the needle was withdrawn and we applied pressure to the wound for 2 minutes to avoid bleeding. The pinhole was covered with a sterile adhesive bandage for 2 days.

The procedure of US-guided MSN release was similar to that of steroid injection except that we used inplane method instead. After skin antisepsis, a 25-gauge needle was introduced and about 2.0 mL of 1% lidocaine was infiltrated into the skin and both superficial and deep layers of the transverse carpal ligament by out-of-plane approach. We placed the transducer around the distal wrist crease to observe the carpal tunnel along its longitudinal axis. When the longitudinal section of the median nerve was detected, tilt the probe slightly toward the ulnar until the median nerve section just disappeared. Then a sterilized MSN (Hanzhang miniscalpel-needle, Huaxia Meditech 53 Inc., Beijing, China) was used to release the carpal tunnel [17]. The MSN was inserted 30° to the skin and along the ulnar side of the median nerve, with the bevel of the MSN parallel to the long axis of hand (Figure 1(c)). The MSN shaft is seen as a hyperechoic bright line in the long axis under ultrasound (Figure 1(b)). Release was performed by moving the MSN forwards and backwards through the transverse carpel ligament for 10-15 times and gradually adjusting the needle tip from proximal to distal so that the carpel tunnel is fully decompressed. After that, the MSN was withdrawn (Figure 1(d)) and 1.0 ml of compound betamethasone (2 mg betamethasone sodium phosphate and 5 mg betamethasone dipropionate) was injected. At last, pressure was applied to avoid bleeding and the minimally invasive wound was covered with a sterile adhesive bandage for 2 days.

After treatment, patients in both groups were observed for 30 minutes to record any adverse reaction. In this study, the US-guided operations were performed using an ultrasound device (CHISON Q9, CHISON Medical Imaging Co., Ltd, Jiangsu province, China, with an 8-12 MHz liner array probe).

### 2.3. Outcome Measures

To estimate the efficacy of the two treatment regimens, patients from both groups were asked to complete Boston Carpal Tunnel Questionnaire and accept a series of tests including four electrophysiological parameters and cross-sectional area of the median nerve at baseline, 4 and 12 weeks after treatment. All the staff collecting outcome data were blinded to the group assignment.

### 2.4. Boston Carpal Tunnel Questionnaire

Boston Carpal Tunnel Questionnaire (BCTQ) consists of two multi-item scales: the Symptom Severity Scale (SSS) and the Functional Status Scale (FSS), which can be used to assess the severity of symptoms and functional status. The SSS evaluates symptoms like numbness, pain, and weakness. The FSS evaluates difficulties with daily activities like writing, buttoning clothes, and gripping a telephone handle. Each score is calculated as the mean of the responses of the individual items [26].

### 2.5. Electrophysiological Outcome

Electrophysiological studies were performed by a standard method using a Medtronic Keypoint EMG Unit [27]. Compound muscle action potential (CMAP) was obtained by placing the active recording electrode on the abductor pollicis brevis muscle belly and the reference electrode on the tendon. The median nerve was stimulated 14cm proximal to the active recording electrode. Distal motor latency (DML) was measured from the onset of stimulus artifact to the onset of the CMAP. Sensory nerve action potential (SNAP) was obtained using an orthodromic method and recorded by surface electrodes placed at the distal radioulnar joint. The median nerve was stimulated at the proximal of the middle finger. The sensory nerve conduction velocity (SNCV) was calculated by dividing the distance by the distal sensory latency.

### 2.6. Cross-Sectional Area of the Median Nerve

An 8-12 MHz linear array transducer (CHISON Q9, CHISON Medical Imaging Co.Ltd, Jiangsu province, China) was used to measure the cross-sectional area (CSA) of the median nerve. The CSA of the median nerve was assessed at the carpal tunnel inlet (the scaphoid-pisiform level) during transverse scanning [28–30]. Examinations before and after treatment were performed in the same standardized manner.

### 2.7. Statistical Methods

Statistical analysis was performed using SPSS 21.0 software (SPSS Inc., Chicago, IL). The comparison of baseline data between the two groups was evaluated by* t*-test for parametric data and by chi-squared test for categorical data. All of the baseline characteristics were adequately normally distributed and the two population variances were equal at the significant level 0.10. Sphericity assumption was identified by Mauchly's Sphericity test. We used the ANOVA for repeated measures to analyze the interaction between treatment effect and time effect, their main effects, and simple effects. Statistical significance was assumed if* P *< 0.05.

## 3. Results

After screening by inclusion and exclusion criteria, a total of 51 patients (51 wrists) with carpal tunnel syndrome were included and randomly assigned into 2 groups, with 25 patients (25 wrists) in steroid injection combined with MSN release (Group A) and 26 patients (26 wrists) in steroid injection (Group B). Finally, due to loss to follow-up (2 patients in Group A and 3 patients in Group B), 23 patients in Group A and 23 patients in Group B completed the 12-week follow-up. The average age of Group A was 48.7±15.2 and the average age of the Group B was 53.1±14.6. All of the baseline characteristics (age, sex, body mass index, and symptom duration) (Table 1) and different parameters of carpal tunnel syndrome (Table 2) showed no statistical difference at baseline between the two groups (*P*>0.05).

### 3.1. Changes of Boston Carpal Tunnel Questionnaire (BCTQ)

Since the interaction effect between time and group is statistically significant for both Symptom Severity Scale (SSS) (*P=*0.002) and Functional Status Scale (FSS) (*P=*0.001), we mainly consider the simple effect. Compared with the baseline values, significant symptom relief in both groups was detected at week 4 and week 12, respectively (*P*<0.001). The FSS also showed statistically significant improvement in both groups at week 4 and week 12, respectively (*P*<0.001). Furthermore, when compared with Group B, Group A showed a statistically better outcome in SSS (*P*=0.001) and FSS (*P*=0.004) at week 12 after treatment (Table 2).

### 3.2. Changes of Compound Muscle Action Potential (CMAP)

By using the ANOVA for repeated measures, we found that there was a statistically significant interaction effect between time and group (*P<*0.001). At week 4 after treatment, CMAP already showed statistically significant improvement in both groups (*P<*0.001) and this statistical difference still existed in week 12 (*P<*0.001). At week 12 after treatment, the treatment effect of Group A was obviously better than that of Group B (*P=*0.024) (Table 2).

### 3.3. Changes of Distal Motor Latency (DML)

As a result of the ANOVA for repeated measures carried out on DML, there was a significant difference in accordance with group effect (*P*=0.002) and time effect (*P*<0.001) and no interaction effect between time and group was found (*P*=0.910) (Table 2).

### 3.4. Changes of Sensory Nerve Action Potential (SNAP)

**Figure 2:**
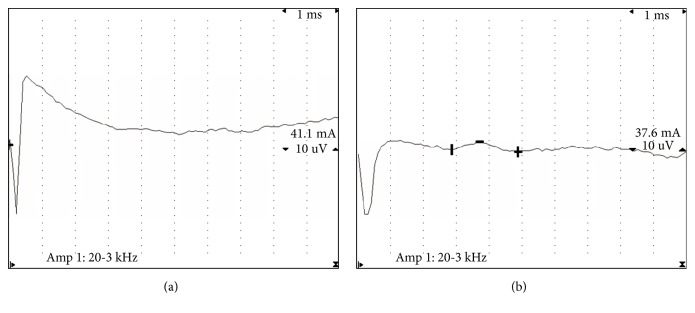
The sensory nerve conduction tests in a 54-year-old woman who received combined therapy of steroid injection and MSN release. (a) The SNAP was not detected before treatment. (b) Four weeks after treatment, the SNAP could be detected although with low amplitude.

SNAP was evaluated at baseline, week 4 and week 12. No interaction effect between time and group was found (*P*=0.691). There was a significant difference in accordance with time main effect (*P*<0.001). But no significant differences were found between the two groups (*P*=0.368) (Table 2). A patient received combined therapy of steroid injection and MSN release. Initially, the SNAP was not detected (Figure 2(a)). Four weeks after treatment, the SNAP could be detected although with low amplitude (Figure 2(b)).

### 3.5. Changes of Sensory Nerve Conduction Velocity (SNCV)

There was a statistically significant interaction effect between time and group (*P=*0.017). When compared with baseline, SNCV showed statistically significant improvement in both groups at week 4 and week 12 after treatment (*P<*0.001). When the two groups were compared, the efficacy of Group A was significantly superior to that of Group B at week 12 (*P=*0.035) (Table 2).

### 3.6. Changes of Cross-Sectional Area of the Median Nerve (CSA)

There was a statistically significant interaction effect between time and group (*P=*0.001). Similarly, at week 4 and week 12 after treatment, CSA showed statistically significant improvement in both groups compared with baseline (*P*<0.05). Furthermore, the efficacy of Group A was significantly superior to that of Group B only at week 12 (*P=*0.028) (Table 2).

Complications, such as nerve injuries or infections, were not observed in either group. One patient (one wrist) in Group A had mild pain after MSN release, but the pain had disappeared within 24 hours. There was no significant difference between the two groups in terms of side effects (*P*>0.05) (data not shown).

## 4. Discussion

To our knowledge, this is the first randomized controlled trial to explore the effectiveness of steroid injection combined with MSN release under ultrasound guidance in treating carpal tunnel syndrome. The results showed that all the parameters in both groups had statistically significant improvement at week 4 and week 12 follow-up compared with baseline. Compared with steroid injection group, the outcomes including BCTQ, DML, CMAP, SNCV, and CSA of the median nerve were statistically better in steroid injection combined with MSN release group at week 12 after treatment. The differences of these parameters between the two groups were close to those in previous studies [29, 31–33]. However, previous studies exploring the minimal clinically important differences (MCID) for the Boston Carpal Tunnel Questionnaire showed that an absolute value of 1.14 point change in the SSS and 0.74 point change in the FSS indicated a clinically relevant threshold of satisfaction while the MCID was 0.46 for SSS and 0.28 for FSS when relative changes were considered [34, 35]. In our study, the differences before and after treatment were consistent with MCID, but the differences between the two groups were smaller than MCID. Since simple steroid injection is a routine treatment, the differences between combined therapy and steroid injection alone may not be particularly significant in the short term. In addition, our limited sample size and the differences in severity of patients may also contribute to the absence of significant clinical differences in BCTQ. During the 3-month follow-up, we found that one patient in Group B did not recover well and underwent surgery 3 months after steroid injection while the conditions of patients in Group A were significantly improved. Overall, we believe that both of the two groups have significant improvement after treatment. At week 12, the combined therapy group showed statistically better results than simple steroid injection group, but larger samples and longer follow-up are needed to achieve more significant clinical differences.

Steroid injection is effective mainly through its local anti-inflammatory mechanism [7]. But Hui, A C et al. [9] and Celik, G et al. [10] found that the long-term effect of steroid injection is not good enough and some patients cannot avoid surgery or multiple injections eventually. Therefore, sometimes, it is still necessary to loosen the transverse ligament of wrist so as to reduce compression mechanically. But surgery release is often rejected by patients due to its scar retention and complexity. In addition to release by surgery, percutaneous release, which is minimally invasive and cost effectively, has been performed and several techniques using different instruments have been reported with satisfactory results. McShane JM et al. reported that 17 patients with a clinical diagnosis of carpal tunnel syndrome had undergone an ultrasonically guided percutaneous needle release and patients had significant symptomatic and functional improvement [29]. In addition, Markison, R E [36] used a technique MANOS to release carpal tunnel. Guo, D et al. [1] released carpal tunnel using a piece of thread looped percutaneously under the visualization of ultrasound. In China, MSN treatment has been developed for many years and is being increasingly used for a variety of pain conditions, including plantar fasciitis, cervical myofascial pain syndrome, trigger thumb, and tendonitis [12–17]. MSN is shaped like an acupuncture needle with a flat and blunt edge on the tip. When using MSN to release carpal tunnel, it can effectively relieve the pressure in carpal tunnel, thereby reducing the compression of the median nerve. In the meantime, this local release may also lead to better diffusion and absorption of the steroid injected afterwards. Furthermore, since it is structurally similar to traditional acupuncture needles, it can both mechanically release transverse carpal ligament and relieve pain through the principles of acupuncture therapy [17]. Based on these advantages, in our study, the combined therapy showed a significantly better therapeutic efficacy than simple steroid injection at week 12 in terms of BCTQ, CMAP, DML, SNCV, and CSA.

Steroid injection has a good short-term effect. Gelberman et al. reported that the maximal improvement in symptoms occurred 1 month to 2 months after steroid injection [37]. So at week 4 in our study, although all the evaluation parameters of the two groups were significantly improved compared with the baseline, there was no significant difference between the two groups. This might be due to the powerful role of steroid injection in the early stages of the two groups, which covered the effect of MSN release to certain degree. However, after 12 weeks of treatment, all the parameters in the combined therapy group, except SNAP, were significantly better than those in steroid injection group. This further illustrates the role of steroid injection combined with MSN release in the long-term effect of treating carpal tunnel syndrome.

Among all the patients involved in this study, no serious complications such as nerve injury occurred during the follow-up period. One possible reason may be due to the blunt edge of MSN, which can avoid direct severing or stabbing the nerve to a certain extent. Another possible explanation could be that we conducted both MSN release and steroid injection under ultrasound guidance in our study. Rojo-Manaute, J M et al. found ultraminimally invasive ultrasound-guided carpal tunnel release provided an earlier functional return and less postoperative morbidity compared with mini-open carpal tunnel release [38]. Ustun, N et al. and Evers, S et al. also found ultrasound-guided steroid injection is superior to blind injection [21, 22]. So, the application of ultrasound is a good technical means for this study. Importantly, since there is no radiation exposure by using ultrasound, patients and practitioners are also likely to accept this assistive technology [39].

Due to the limited follow-up time and sample size, we recommend that additional researches with larger samples and longer follow-up should be conducted in the future in order to achieve more accurate and comprehensive results. In addition, in our study, the treatment execution and return were handled separately. All the staff who collected outcome data were not informed of the group assignment of patients. But due to the nature of the procedures, it was impossible to blind the patients and make the subjective results (SSS and FSS) susceptible to placebo effects. We evaluated the efficacy by means of some electrophysiology and ultrasound indicators and more objective indicators are also needed in future experiments to get more objective results.

## 5. Conclusions

The effectiveness of steroid injection combined with MSN release for CTS is superior to that of steroid injection alone, which may have important implications for future clinical practice.

## Figures and Tables

**Table 1:** Demographic characteristics of the two groups at baseline.

Demographic Characteristics	Group A (n=23)	Group B (n=23)	Significance of Shapiro-Wilk test		Significance of Levene's Test for Equality of Variances	Significance of *t* test or chi-square test
			Group A	Group B		
Age (y)	48.7±15.2	53.1±14.6	0.397	0.329	0.968	0.323
Sex (female/male)	18/5	17/6				0.730
Body mass index (kg/m^2^)	24.1±1.7	24.7±1.6	0.340	0.599	0.696	0.253
Symptom duration (m)	10.2±3.5	11.1±2.8	0.686	0.565	0.188	0.333

*Note.* Group A was steroid injection combined with MSN release group. Group B was steroid injection group. The demographic characteristics of the two groups at baseline were described using Mean ± Standard Deviation (*SD*). There was no significant difference between Group A and Group B.

**Table 2:** Outcome measurements of BCTQ, DML, CMAP, SNAP, SNCV, and CSA among patients at different time points.

	Baseline	4 weeks	12 weeks		*F*	*P*
*BCTQ-SSS*						
Group A	3.10±0.32	2.34±0.21^*∗∗∗*^	1.84±0.21^*∗∗∗*##^	Group (*G*)	2.892	0.096
Group B	3.00±0.25	2.47±0.25^*∗∗∗*^	2.06±0.23^*∗∗∗*^	Time (*T*)	292.617	<0.001
				G×*T*	6.690	0.002
Mauchly's Sphericity test* W*=0.882 (*P=*0.067)						
*BCTQ-FSS*						
Group A	3.10±0.25	2.46±0.22^*∗∗∗*^	1.80±0.35^*∗∗∗*##^	Group (*G*)	2.634	0.112
Group B	3.00±0.25	2.53±0.24^*∗∗∗*^	2.08±0.27^*∗∗∗*^	Time (*T*)	235.033	<0.001
				G×*T*	7.144	0.001
Mauchly's Sphericity test* W*=0.987 (*P=*0.754)						
*CMAP (mV) *						
Group A	9.4±1.2	9.7±1.2^*∗∗∗*^	12.2±1.3^*∗∗∗*#^	Group (*G*)	0.259	0.613
Group B	9.5±1.1	9.9±1.2^*∗∗∗*^	11.3±1.1^*∗∗∗*^	Time (*T*)	462.702	<0.001
				G×*T*	26.119	<0.001
Mauchly's Sphericity test* W*=0.902 (*P=*0.110)						
*DML (ms)*						
Group A	5.2±0.3	4.9±0.3^*∗∗∗*^	4.5±0.4^*∗∗∗*#^	Group (*G*)	11.214	0.002
Group B	5.4±0.3	5.1±0.3^*∗∗∗*^	4.7±0.4^*∗∗∗*^	Time (*T*)	51.852	<0.001
				G×*T*	0.094	0.910
Mauchly's Sphericity test* W*=0.899 (*P=*0.100)						
*SNAP (μV)*						
Group A	12.1±1.8	14.1±2.6^*∗∗*^	16.3±3.5^*∗∗∗*^	Group (G)	0.829	0.368
Group B	12.0±1.6	13.5±2.7^*∗*^	15.4±2.7^*∗∗∗*^	Time (T)	34.857	<0.001
				G×T	0.371	0.691
Mauchly's Sphericity test *W*=0.963 (*P*=0.448)						
*SNCV (m/s) *						
Group A	38.6±3.8	42.2±2.8^*∗∗∗*^	46.5±2.5^*∗∗∗*#^	Group (*G*)	0.283	0.597
Group B	39.5±3.2	42.1±2.2^*∗∗∗*^	44.7±3.2^*∗∗∗*^	Time (*T*)	99.794	<0.001
				G×*T*	4.292	0.017
Mauchly's Sphericity test *W*=0.909 (*P*=0.128)						
*CSA (mm* ^*2*^)						
Group A	13.3±1.4	12.5±1.4^*∗∗∗*^	10.8±1.1^*∗∗∗*#^	Group (*G*)	0.477	0.493
Group B	13.1±1.5	12.7±1.4^*∗*^	11.6±1.2^*∗∗∗*^	Time (*T*)	108.913	<0.001
				G×*T*	7.251	0.001
Mauchly's Sphericity test* W*=0.969(*P*=0.503)						

*Note.* Group A was steroid injection combined with MSN release group. Group B was steroid injection group. Values were described using Mean ± Standard Deviation (*SD*). Comparisons between week 4 follow-up and baseline and week 12 follow-up and baseline, respectively. ^*∗*^ *P *< 0.05, ^*∗∗*^ *P *< 0.01, and ^*∗∗∗*^ *P *< 0.001. Comparisons between group A and group B at corresponding time points. ^#^ *P *< 0.05, ^##^ *P *< 0.01, and ^###^ *P *< 0.001. *Abbreviations*. BCTQ-SSS: the symptom severity scale of Boston carpal tunnel questionnaire; BCTQ-FSS: the functional status scale of Boston carpal tunnel questionnaire; CMAP: compound muscle action potential; DML: distal motor latency; SNAP: sensory nerve action potential; SNCV: sensory nerve conduction velocity; CSA: cross-sectional area of the median nerve.

## Data Availability

The data used to support the findings of this study are included within the article.
